# Transient constrictive pericarditis following coxsackievirus A4 infection as a rare cause of acute mediastinitis: A case report

**DOI:** 10.1016/j.heliyon.2023.e19555

**Published:** 2023-08-30

**Authors:** Hiroyuki Yamamoto, Jun Isogai

**Affiliations:** a Department of Cardiovascular Medicine, Narita-Tomisato Tokushukai Hospital, Chiba, Japan; b Division of Radiology, Asahi General Hospital, Asahi, Japan

**Keywords:** Transient constrictive pericarditis, Acute mediastinitis, Coxsackievirus A4, Constrictive physiology, Cardiac magnetic resonance, Anti-inflammatory treatment

## Abstract

**Background:**

Transient constrictive pericarditis (TCP) is a distinct constrictive pericarditis (CP) subtype characterized by acute pericardial inflammation and transient constrictive physiology. If left untreated, it may progress to irreversible CP requiring pericardiectomy. However, making an early diagnosis of TCP remains difficult.

**Case presentation:**

A 51-year-old man presented with fever, chest pain, and dyspnea following preceding flu symptoms. An initial investigation suggested right-sided heart failure. Laboratory results revealed elevated inflammatory markers and hepatic enzyme levels. Echocardiography revealed pericardial effusion with a normal ejection fraction and diastolic ventricular septal bounce suggestive of pericardial constriction. Computed tomography suggested acute descending mediastinitis with pericarditis and pleuritis; however, detailed examinations ruled out this possibility. The constellation of increased serological inflammation, pericardial thickness/effusion, and constrictive physiology suggested TCP, confirmed by cardiac magnetic resonance (CMR) and hemodynamic studies. CMR also revealed coexistent myocarditis. After a thorough assessment for the cause of TCP, a viral etiology was suspected. Paired serology for virus antibody titers revealed a significant increase only in coxsackievirus A4 (CVA4) titers. With prompt anti-inflammatory treatment, the patient’s pericardial structure and function and concomitant inflammation of the surrounding tissues were nearly completely recovered, leading to a final diagnosis of TCP caused by CVA4. The subsequent clinical course was uneventful without recurrence at the 1-year follow-up.

**Conclusions:**

Here we described the first case of TCP caused by CVA4 concurrent with mediastinitis, myocarditis, and pleuritis, all of which were successfully resolved with anti-inflammatory treatment. Acute mediastinitis secondary to TCP is rare. This case highlights the clinical importance of assessing pericardial diseases as a source of acute mediastinitis and considering CVA4 as an etiology of TCP. An evaluation including multimodal cardiac imaging and serology for virus antibody titers may be useful for an exploratory diagnosis of TCP in right-sided heart failure patients with pericardial effusion.

## 1 Introduction

### 1.1 Background

Constrictive pericarditis (CP) is a potentially fatal disorder characterized by inflammation and subsequent fibrosis of the pericardium resulting in loss of pericardial distensibility that manifests as signs and symptoms of right-sided heart failure based on impaired diastolic cardiac filling and requiring pericardiectomy [1]. CP is classified into three distinct subtypes: transient, effusive, and chronic. Transient constrictive pericarditis (TCP), which accounts for 9–17% of all CP cases [2,3], is a specific CP subtype characterized by pericardial thickening, pericardial effusion, and transient constrictive physiology that resolve spontaneously or respond to anti-inflammatory treatment [2]. The pathophysiology underlying TCP is a transient loss of pericardial elasticity secondary to inflammation, edema, and fibrin deposition in the pericardium, but is quite different from that of chronic CP, in which a permanent loss of pericardial elasticity caused by pericardial fibrosis and calcification is observed [3,4]. Effusive constrictive pericarditis (ECP) is another CP subtype characterized by pericardial effusion that potentially causes pericardial tamponade and advanced constrictive physiology; however, its clinical signs may overlap with those of TCP. Therefore, making an accurate diagnosis of TCP remains a challenge. Here we describe a first adult case of TCP caused by a coxsackievirus A4 (CVA4) monoinfection mimicking acute descending mediastinitis. Multimodal cardiac imaging aided its accurate diagnosis.

## 2 Case presentation

A 51-year-old Scottish man was admitted to our hospital with fever, substernal chest pain, and dyspnea after flu symptoms 10 days prior. He had no recent history of esophagogastroduodenoscopy manipulation, cardiac surgery, or chest trauma. Physical examination findings were as follows: blood pressure, 128/90 mmHg; high-grade fever, 38.8 °C; heart rate, 117 beats/min; and oxygen saturation, 94% on ambient air. Jugular vein distention, diminished breath sounds from the left lower lung, and mild bilateral leg edema were noted. The rest of the physical examination, including the oral cavity and mucocutaneous examination, was unremarkable.

Laboratory test results revealed leukocytosis (13,100/μL) with neutrophilia of 82.6% and elevated levels of N-terminal pro-brain natriuretic peptide (235 pg/mL; normal, <125 pg/mL) and liver enzymes (aspartate aminotransferase, 43 U/L; normal, 13–30 U/L; alanine aminotransferase, 90 U/L; normal, 7–23 U/L). Inflammatory marker levels were also elevated: C-reactive protein (CRP), 20.31 mg/dL (normal, <0.3 mg/dL); erythrocyte sedimentation rate, >110 mm/h (normal, 3–15 mm/h); procalcitonin, 0.09 ng/mL (normal, <0.05 ng/mL); and pro-inflammatory cytokine-related interleukin-6 (IL-6), 157 pg/mL (normal, <7.0 pg/mL). Screening for thyroid and autoimmune diseases and *Mycobacterium tuberculosis* infection yielded negative results. In addition, the serological screening tests for hepatitis B, hepatitis C, human T-cell leukemia virus type 1, and human immunodeficiency viruses (types 1 and 2) were negative (Table 1).

**Table 1:** Serological tests for viral infections. Changes in serum viral antibody titer of Coxsackievirus A4 showing an 8-fold increase (bold font). CLEIA, chemiluminescent enzyme immunoassay; EA-DR, Epstein-Barr virus-early antigen, diffuse type and restricted type antibody; EIA, enzyme immunoassay; ELISA; enzyme-linked immuno sorbent assay; FA, direct immunofluorescence assay; HBsAg, hepatitis B virus-specific antigen; HCV, hepatitis C virus; HI, hemagglutination inhibition test; HIV, human immunodeficiency virus; HTLV, human T-cell leukemia virus; NT, neutralization; VCA, viral capsid antigen.

Table 1. Serological tests for viral infections									
Virus	Type	Method	Titer on admission	Titer on day 21	Virus	Type	Method	Titer on admission	Titer on day 21
Adenovirus	1	NT	–	8	Echovirus	1	NT	–	<4
	2	NT	–	4		3	NT	–	<4
	3	NT	–	<4		4	NT	–	<4
	4	NT	–	<4		5	NT	–	<4
	5	NT	–	4		6	NT	–	4
	6	NT	–	<4		7	NT	–	<4
	7	NT	–	4		9	NT	–	<4
	8	NT	–	<4		11	NT	64	128
	11	NT	–	<4		12	NT	–	<4
	19	NT	–	<4		13	NT	–	<4
	21	NT	–	<4		14	NT	–	8
	37	NT	–	<4		16	NT	32	32
Coxsackievirus	A2	NT	8	16		17	NT	–	4
	A3	NT	–	<4		18	NT	–	<4
	**A4**	**NT**	**64**	**512**		19	NT	32	32
	A5	NT	64	128		21	NT	–	<4
	A6	NT	32	16		22	NT	64	64
	A7	NT	8	16		24	NT	–	4
	A9	NT	–	4		25	NT	–	8
	A10	NT	–	<4		30	NT	–	<4
	A16	NT	32	16	Influenza virus	A (H1N1)	HI	–	<10
	B1	NT	16	16		A (H3N2)	HI	–	20
	B2	NT	–	8		B1	HI	–	20
	B3	NT	–	4		B2	HI	80	160
	B4	NT	–	4	Parainfluenza virus	1	HI	–	<10
	B5	NT	–	8		2	HI	–	10
	B6	NT	–	<4		3	HI	320	320
HBsAg		CLEIA	Negative		Respiratory syncytial virus		NT	8	16
anti-HCV		CLEIA	Negative		Parvovirus	B19	EIA	IgM, 0.24	IgM, 0.28
anti-HTLV-1		ELISA	Negative		Epstein-Barr virus	EA-DR	FA	–	IgG, <10
anti-HIV-1		CLEIA	Negative			VCA		IgM, 0.24	IgM, 0.24
anti-HIV-2		CLEIA	Negative					–	IgG, 40

**Fig. 1:**
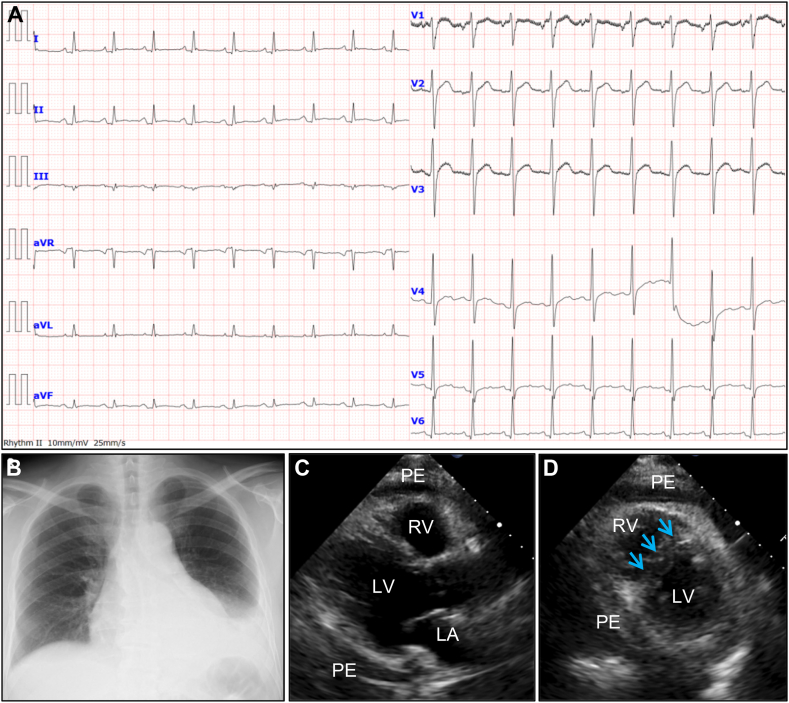
Initial screening tests An initial electrocardiogram revealing sinus tachycardia (heart rate, 113 beats/min), generalized T-wave flattening, and PR-segment depression (A). Note the reciprocal PR-segment elevation in lead aVR. Chest radiography reveals an enlarged flask-shaped cardiac silhouette and left-side pleural effusion without enlargement of the pulmonary vascular shadow (B). Initial transthoracic echocardiography (C and D). The parasternal long-axis view (C) revealing a moderate circumferential PE. The parasternal short-axis view of mid-LV level (D) revealing abnormal diastolic septal motion of the interventricular septum (arrows). The ruler presented in this image has divisions 1 cm apart. LA, left atrium; LV, left ventricle; PE, pericardial effusion; RV, right ventricle.

Electrocardiogram (ECG) showed sinus tachycardia, widespread T-wave flattening, and PR-segment depression. Reciprocal PR elevation was noted in lead aVR suggestive of pericarditis (Fig. 1A). Chest radiography revealed cardiomegaly and left-sided pleural effusion without pulmonary congestion (Fig. 1B). Echocardiography showed moderate circumferential pericardial effusion, a plethoric inferior vena cava, and marked diastolic ventricular septal shift but no signs of cardiac tamponade, suggesting high right-sided pressures with constrictive physiology (Fig. 1C and D, and Video S1 in the Data Supplement).

**Fig. 2:**
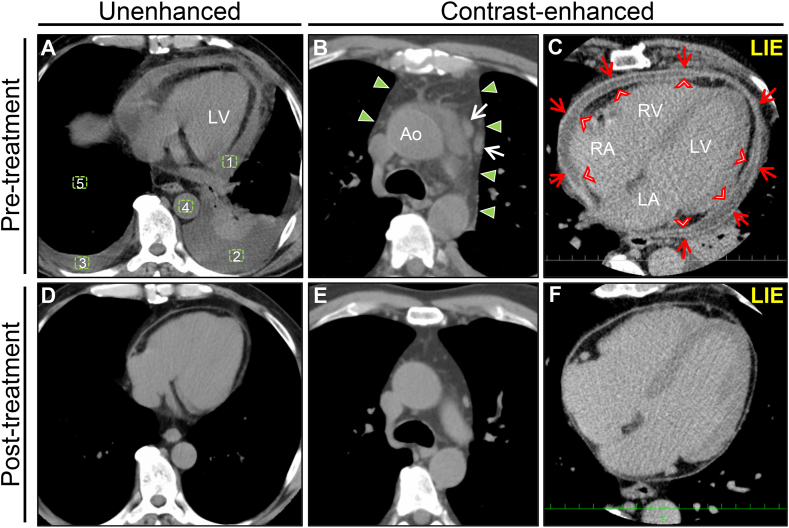
Effects of anti-inflammatory treatment on CT Unenhanced CT and electrocardiogram-gated contrast-enhanced CCT findings on admission (A–C) and after 2 months of treatment (D–F). Pre-treatment chest CT revealing a pericardial effusion and bilateral pleural effusions with left lower lobe atelectasis (A). We measured each CT attenuation value corresponding to each box shown in Fig. 2A as follows: box 1, 21.8 HU; box 2, 12.1 HU; box 3, -3.7 HU; box 4, 35.7 HU; and box 5, -830.6 HU. Post-treatment chest CT revealing complete resolution of the abnormal CT findings observed on admission (D). Pre-treatment CCT revealing increased attenuation with fat stranding and enlargement of the superior mediastinum (arrowheads) at the level of the aortic arch (B). Note the enlarged periaortic lymph nodes (arrows). Post-treatment CCT showing complete resolution of these abnormal findings (E). Pre-treatment LIE image revealing diffuse pericardial thickening on the parietal and visceral sides (red arrows and red arrowheads, respectively) observed on admission (C), all of which resolved completely on the post-treatment LIE image (F). The ruler presented in this image has divisions 1 cm apart. Ao, aorta; CCT, cardiac computed tomography; CT, computed tomography; HU, Hounsfield unit; LIE, late iodine enhancement; LA, left atrium; LV, left ventricle; RA, right atrium; RV, right ventricle.

Unenhanced chest computed tomography (CT) revealed mediastinal enlargement, pericardial effusion, and bilateral pleural effusions. CT also showed elevated attenuation values for the pericardial and pleural effusions suggestive of an infectious etiology (Fig. 2A). Detailed ECG-gated cardiac computed tomography (CCT) revealed mediastinal enlargement associated with superior mediastinal fat stranding and periaortic enlarged lymph nodes strongly suggestive of acute mediastinitis (Fig. 2B). A late iodine enhancement (LIE) image showed prominent enhancement of the entire pericardium and epicardium without pericardial calcification, suggesting acute pericardial inflammation (Fig. 2C). Accordingly, the patient was presumptively diagnosed with acute descending mediastinitis, pleuritis, and pericarditis. Empirical antimicrobial treatment with intravenous ceftriaxone (1 g/12 h) and clindamycin (300 mg/6 h) was initiated. A thorough examination of the specific cause of acute mediastinitis was performed (head and neck CT, esophagogastroduodenoscopy) and all findings were normal; therefore, we excluded acute descending mediastinitis from the differential diagnosis.

**Fig. 3:**
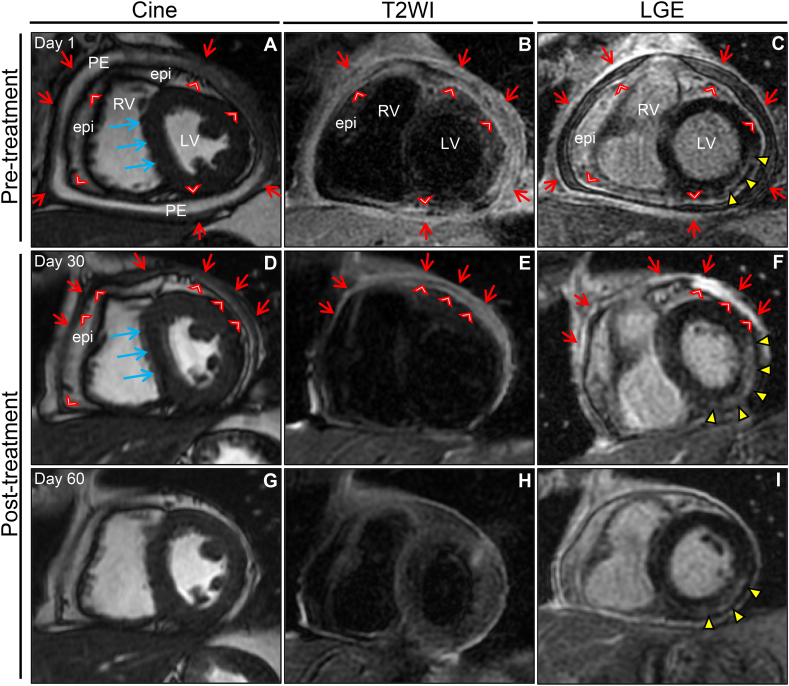
Effects of anti-inflammatory treatment on CMR The cine image shows marked thickening of the entire pericardium and epicardium surrounding the heart (parietal pericardium, red arrow; visceral pericardium, red arrowheads) with a moderate PE and an evident diastolic ventricular septal bounce (blue arrows) on admission (A). Pericardial functional and structural abnormalities persisted despite resolution of the PE after 1 month of treatment (D), which resolved clearly after 2 months of treatment (G). A T2WI image shows diffuse pericardial edema (parietal pericardium, red arrows; visceral pericardium, red arrowheads) observed on admission (B), which persisted after 1 month of treatment (E) and resolved completely after 2 months of treatment (H). LGE image taken on admission (C) shows moderate enhancement of the pericardium (parietal pericardium, red arrows; visceral pericardium, red arrowheads) and partial further intensification (as shown by a bright thick white line) after 1 month of treatment (F) but resolved completely after 2 months of treatment (I). In addition, the LGE in the subepicardial lateral wall of the LV at the papillary muscle level (yellow arrowheads) observed on admission (C) showed further transmural enhancement after 1 month of treatment (F) and remained partially without pericardial LGE after 2 months of treatment (I). CMR, cardiac magnetic resonance; epi, epicardial fat; LGE, late gadolinium enhancement; LV, left ventricle; PE, pericardial effusion; RV, right ventricle; T2WI, T2-weighted image.

**Fig. 4:**
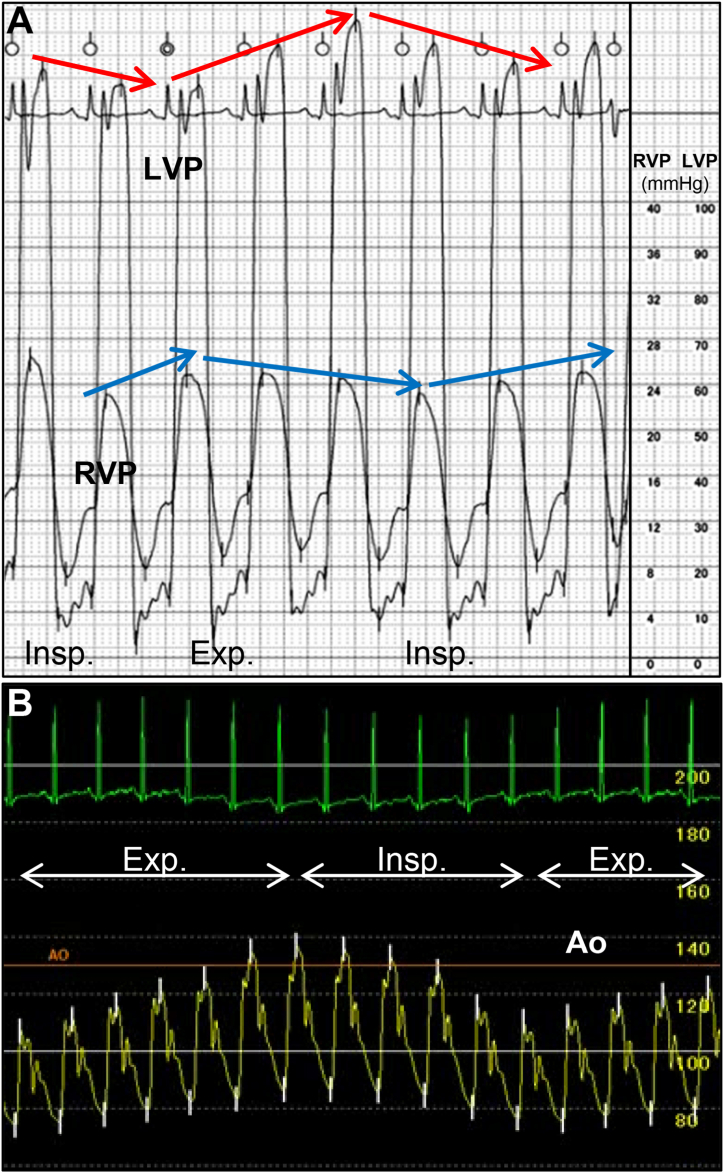
Hemodynamic evaluation (A) Simultaneous right and left ventricular pressure waveforms revealing a square root sign during diastole with ventricular interdependence. Note the discordance of the right and left ventricular systolic pressures. (B) An aortic blood pressure waveform revealed a significant inspiratory drop in systolic pressure of greater than 40 mmHg, indicating prominent pulsus paradoxus. Ao, aorta; Exp., expiration; Insp., inspiration; LVP, left ventricular pressure; RVP, right ventricular pressure.

The constellation of increased serological inflammation, thickened pericardial inflammation, and constrictive physiology was highly suggestive of TCP. Cardiac magnetic resonance (CMR) imaging further characterized the pericardial inflammation (Fig. 3A–C, and Video S2 in the Data Supplement). The cine mode showed moderate pericardial effusion and abnormal diastolic ventricular septal bounce with thickening of the entire pericardium showing high signal intensity on fat-suppressed T2-weighted images (T2WI). A late gadolinium enhancement (LGE) image showed moderate enhancement in the pericardium consistent with acute pericarditis. LGE also revealed myocardial involvement beneath the epicardium in the lateral wall suggestive of coexisting myocarditis. Hemodynamics in cardiac catheterization confirmed the characteristic findings of a constrictive physiology (Fig. 4A and B).

Subsequent oral anti-inflammatory treatment with ibuprofen (200 mg 3 times daily) and colchicine (0.5 mg orally 2 times daily) was initiated. A vasopressin V2-receptor antagonist (tolvaptan 7.5 mg/day), loop diuretics (azosemide 30 mg/day), and a mineralocorticoid receptor antagonist (spironolactone 50 mg/day) were added to ameliorate the systemic congestion. All pleural fluid and repeated blood cultures were negative. Pleural fluid cytology was negative for malignancy, and the antimicrobials were stopped. Thereafter, the patient’s condition improved steadily and he was discharged on day 7 after admission.

One week later, positron emission tomography CT showed marked improvement in the pericardial and pleural effusions yet residual slightly hypermetabolic activity in the pericardium and left pleura (Fig. S1A in the Data Supplement). On day 21, antibody titer blood tests for CVA4 revealed an 8-fold increase, from 64 to 512, but no significant changes were detected for the other viruses, suggesting an acute CVA4 monoinfection (Table 1). Therefore, we diagnosed the patient with acute pericarditis and inflammation spreading to the surrounding tissues caused by CVA4. Follow-up CMR imaging performed on day 30 showed further exacerbation of the myocardial lesions, somewhat stronger enhancement of the pericardial LGE, and residual constrictive physiology despite resolution of the pericardial effusion (Fig. 3D–F). However, the patient's clinical condition remained improved with good biochemical responses (CRP, 2.27 mg/dL; IL-6, 50 pg/mL) and normalized hepatic dysfunction, suggesting that they could reflect an inflammatory response due to CVA4 infection and systemic congestion due to right-sided heart failure, respectively. Therefore, the anti-inflammatory treatment was continued whereas the anti-heart failure treatment was discontinued.

Finally, CMR performed on day 60 showed significant improvement in the myocardial lesions and complete recovery of the pericardial structure and function (Fig. 3G–I, and Video S3 in the Data Supplement). Additionally, CT and CCT confirmed the complete resolution of all abnormal findings observed on admission (Fig. 2D–F). Similarly, the chest X-ray, ECG, and echocardiography findings were normal (Figs. S1B and S1C, and Video S4 in the Data Supplement). This resulted in the final diagnosis of TCP caused by CVA4 infection and associated mediastinitis, myocarditis, and pleuritis.

**Fig. 5:**
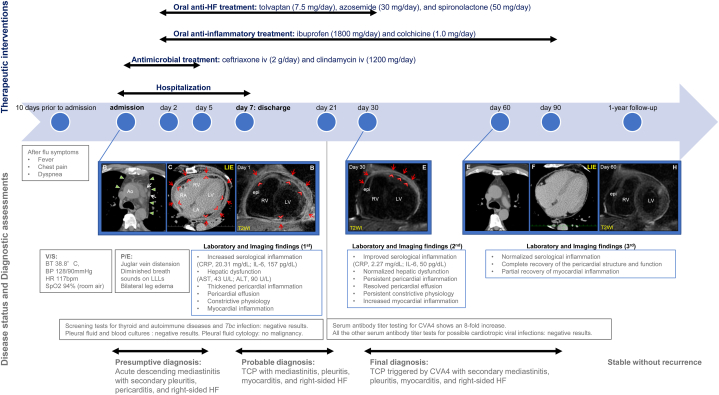
Timeline of the diagnostics, treatment, and disease status of the present case. Abbreviations: Ao, aorta; ALT, alanine aminotransferase; AST, aspartate aminotransferase; BP, blood pressure; BT, blood temperature; CVA4, coxsackievirus A4; CRP, C-reactive protein; epi, epicardial fat; HF, heart failure; HR, heart rate; IL-6, interleukin-6; LIE, late iodine enhancement; LA, left atrium; LLLs, left lower lobes; LV, left ventricle; P/E, physical examination; RA, right atrium: RV, right ventricle; T2WI, T2-weighted image; TCP, transient constrictive pericarditis; Tbc, tuberculosis; V/S, vital signs.

One month later, the medication was discontinued. The patient remained clinically stable without recurrence of constrictive physiology during the 1-year follow-up period. The timeline comprising diagnostics, treatment, and disease status is shown in Fig. 5.

## 3 Discussion

Although TCP occurs in approximately 15% of acute pericarditis cases, its exact incidence remains unknown due to a lack of awareness [2]. The most frequent causes of TCP in developed countries include connective tissue disorders, idiopathic/viral pericarditis, and cardiac surgery [3,5]. Since the mediastinum and pericardial sac are structurally continuous, some case reports have shown contiguous inflammation from the mediastinum to the pericardial sac due to gravity [[6], [7], [8], [9]], suggesting acute descending mediastinitis as a rare cause of TCP. However, little is known about the reverse relationship. Here we described the first adult case of TCP caused by CVA4 infection associated with myocardial, pleural, and mediastinal involvement, all of which were successfully treated with anti-inflammatory agents. Our case offers four important clinical lessons.

First, this case exhibited atypical TCP, which was initially misdiagnosed as acute descending mediastinitis based on the patient’s symptoms and initial CT findings. Acute mediastinitis is a potentially life-threatening serious inflammation of the mediastinum with an infectious or non-infectious etiology that commonly results from cardiothoracic surgery; followed by esophageal perforation (iatrogenic or idiopathic); the direct spread of pharyngeal, odontogenic, or cervical infections; or, rarely, hematogenous spread [[10], [11], [12], [13], [14]]. Its symptoms are similar to those of pericarditis, including chest pain, dyspnea, and fever.

The early diagnosis and investigation of the specific cause of mediastinitis are essential, as the catastrophic nature of the disease often requires prompt antimicrobial treatment and surgical debridement. After ruling out all possible causes of acute descending mediastinitis, the patient’s mediastinitis findings on CT disappeared along with the recovery of TCP with anti-inflammatory treatment. Therefore, we concluded that acute ascending mediastinitis resulting from contiguous inflammation from the pericardial sac to the mediastinum was the most likely cause in our case. Our case highlights the clinical importance of pericardial disease as a possible cause of acute mediastinitis.

Second, this is the first adult case of a single CVA4 infection that caused TCP associated with mediastinitis, myocarditis, and pleuritis. The possible etiologies of pericarditis include idiopathic causes (predominantly viral), suppurative and autoimmune diseases, tuberculosis, or neoplastic process [15,16]. In this case, the thorough screening of test results, including laboratory tests, paired virology, blood cultures, and thoracoabdominal CT, ruled out the possibility of any conditions other than viral infection. Finally, the diagnosis was established based on the temporal relationship between the preceding flu symptoms and subsequent clinical manifestations of TCP with significant changes in the CVA4 antibody titers.

Coxsackieviruses belong to the family *Picornaviridae* and genus *Enterovirus* of non-enveloped, single-stranded RNA viruses. Coxsackieviruses are classified into two groups: group A (CVA; at least 23 serotypes) and group B (CVB; six serotypes) [17]. Coxsackieviruses generally prevail in the summer, cause viral infections (predominantly in neonates and young children), and are transmitted by the fecal-oral or respiratory droplet route. Common signs of infection in both groups include mild non-specific febrile illnesses, skin rashes, and upper respiratory tract infections. CVB likely infects the pleura, heart, liver, and pancreas, causing pleurodynia, myocarditis, hepatitis, and pericarditis, whereas CVA likely infects the skin and mucous membranes, typically causing herpangina and hand-foot-and-mouth disease [18]. Particularly, CVA4, which was first isolated during the outbreak of poliomyelitis in 1948, causes a variety of epidemiologically important diseases, comprising febrile illness, acute polyradiculoneuritis, acute flaccid paralysis, mucocutaneous lymph node syndrome, bilateral idiopathic retinal vasculitis, myocarditis, herpangina, and hand-foot-and-mouth disease [18,19]. Hence, CVA4 exerts global health issues, and, particularly in China, several outbreaks of febrile disease caused by CVA4 have been reported [20,21]. Although all age groups are susceptible, neonates and young children, who have a weakened immune system; immunocompromised individuals, such as patients with cancer; or those undergoing long-term systemic steroid treatment are considered at high risk for CVA4 infection.

Although CVB is a well-known cardiotropic virus responsible for myopericarditis [22,23], CVA4 also infects the myocardium in a neonatal murine model [21]. Limited case reports of children with other CVA infections causing myocarditis or pericarditis have been documented [24,25]. A retrospective study of 386 children with culture-proven coxsackievirus infections reported one fatal case of acute myocarditis following CVA4 infection, which represented 1% of all children with CVA4 infections [19]. An adult case of myocarditis following CVA4 infection was also reported [26]. There is one anecdotal case report of a young male with acute myopericarditis caused by coinfection with CVA4 and CVB3 [27]. To the best of our knowledge, this is the first adult case of TCP caused by a single infection with CVA4.

Third, multimodal cardiac imaging was helpful in diagnosing TCP and monitoring of treatment response in this case. CMR can evaluate the morphology and characterization of the inflamed pericardium, constrictive physiology, and myocardial involvement, which led to an accurate diagnosis of TCP associated with myocarditis in our case. A study of 25 patients with biopsy-proven CP analyzed by CMR demonstrated that the pericardial LGE was histologically associated with active inflammation, including chronic inflammation, neovascularization, and fibroblast proliferation [28]. Indeed, the presence of pericardial thickening and pericardial LGE provides good diagnostic yield in identifying patients with TCP (sensitivity, 86%; specificity, 80%) [5]. However, the CMR used in our case had two limitations. First, our case showed a time delay between improvement in the patient’s clinical response and CMR imaging findings during the TCP healing process. The improvement in clinical symptoms and serological inflammatory markers proceeded, however, and the LGE of the pericardium and myocardium with the constrictive physiology persisted for 1 month after treatment, although almost all of the abnormal findings had resolved by 2 months after treatment. Our experience with this case suggests that comprehensive judgment and image monitoring are required when the patient’s clinical symptoms improve rather than a routine switch to invasive pericardiectomy or aggressive immunosuppressive treatment, such as corticosteroids, based on image findings alone. Another limitation is that the presence of obvious adjacent inflamed epicardial/pericardial fat and pericardial effusion interfered with the clear depiction of the pericardial lesion on the initial T2WI and LGE images in our case. However, the LIE image on ECG-gated CCT addressed this limitation and clearly depicted delayed enhancement of the visceral and parietal parts of the thickened pericardium, complementing the ambiguous inflammatory pericardial fat findings observed on CMR. Although LIE imaging is a promising alternative to LGE-CMR for characterizing myocardial disease [29], further data accumulation and analyses are needed of pericardial disease on LIE images.

Fourth, in the present case, acute TCP caused by CVA4 was successfully treated with anti-inflammatory agents alone. Treatment of TCP generally follows the acute pericarditis treatment guidelines, although it must be specific to each etiology. Generally, nonsteroidal anti-inflammatory drugs (NSAIDs) with or without colchicine are the first-line agents for most cases of TCP with active inflammation. Importantly, initial treatment of TCP due to idiopathic or viral etiology should not inadvertently include glucocorticoids because of the potential risk of reactivating viral infections or a delayed viral particle clearance from the pericardium with subsequent ongoing inflammation [30,31]. Furthermore, a comparative study of recurrent pericarditis treated with corticosteroids at high doses (1.0 mg/kg/day) versus low doses (0.2–0.5 mg/kg/day) revealed that higher doses of prednisone were associated with severe side effects, recurrences, or hospitalizations (hazard ratio 3.61; 95% confidence interval, 1.96–6.63; and *p* < 0.001) [32]. Therefore, glucocorticoids can be used for acute TCP but only in patients with poor response to the anti-inflammatory treatment or with specific etiology, such as systemic autoimmune or autoinflammatory diseases, and at its lowest effective dose. Pericardiectomy must be considered as the last option for refractory cases of TCP without active inflammation [33]. Here, pericardiotomy was not required as our patient completely recovered following an anti-inflammatory treatment with NSAIDs and colchicine.

A plausible CP spectrum was recently proposed wherein CP progresses chronologically through three stages – TCP, ECP, and chronic CP – depending on the severity of the pericardial inflammation and reversibility of the constrictive physiology [34]. Furthermore, a retrospective study analyzing 212 patients with CP findings on echocardiography demonstrated the possibility of TCP with any etiology other than radiotherapy [3]. Although acute viral pericarditis generally follows a relatively benign course, even patients with coxsackievirus infection who develop subsequent chronic CP requiring pericardiectomy have been reported [25]. Therefore, the early recognition and prompt intervention of treatable TCP may play an important role in preventing the transition to the irreversible phase of CP and avoiding unnecessary pericardiectomy.

Our case report has several limitations. The definitive diagnosis of viral pericarditis is based on histopathological and immunohistological evaluation of the pericardium and the detection of the viral genome using polymerase chain reaction (PCR). In the present case, the anti-inflammatory treatment successfully resolved constrictive physiology; hence, pericardiocentesis and pericardiectomy were not necessary. Eventually, we were not able to show the direct causal relationship between CVA4 infection and TCP development. Second, the etiological diagnostic accuracy of serological titer testing for viral pericarditis is limited. One prospective comparative study of 124 patients with suspected myocarditis reported that virus serology testing was not always associated with the detection of the viral genome in myocardial biopsy tissue using PCR [35]. Thus, routine viral serology is not currently recommended for cases of suspected pericarditis [33]. However, given that serological virus antibody titers usually increase 4-fold or more during acute infection [36], a significant increase in virus antibody titers may support the diagnosis of acute viral pericarditis in some cases [26,37]. Similarly, considering the increased virus antibody titers and the identification of coxsackievirus that prefers to infect the pericardium and myocardium, we believe that acute CVA4 monoinfection most likely might have caused the TCP in the present case. Finally, it underscores the difficulty of etiologic diagnosis in cases of suspected acute viral pericarditis in which pericardial samples might not be available. Thus, in the setting of certain cardiotropic viruses such as coxsackievirus, a significant increase in viral antibody titers may aid in the etiologic diagnosis. This is a concern for future studies and requires further research data for better comprehension.

## 4 Conclusions

Herein, we described the first adult case of TCP caused by a single infection of CVA4 associated with mediastinitis, myocarditis, and pleuritis that almost fully resolved with anti-inflammatory treatment. Multimodal cardiac imaging and paired serology for virus antibody titers were useful for an early diagnosis and treatment of TCP in our case. Here, we highlight the clinical importance of assessing pericardial diseases as a cause of acute mediastinitis and considering CVA4 as an etiology of TCP. Left untreated, TCP may progress to irreversible chronic CP. Therefore, clinicians should recognize this clinical entity and perform a comprehensive evaluation including multimodal cardiac imaging and viral screening using paired serology for virus antibody titers for an exploratory diagnosis of TCP in right-sided heart failure patients with pericardial effusion.

## Ethical statement

*Consent:* The authors confirm that written consent for submission and publication of this case report including images and the associated videos has been obtained from the patient.

## Author contribution statement

All authors listed have significantly contributed to the investigation, development and writing of this article.

## Data availability statement

Data included in article/supplementary material/referenced in article.

Declaration of interest’s statement:

The authors declare no conflict of interest.

## Declaration of competing interest

The authors declare that they have no known competing financial interests or personal relationships that could have appeared to influence the work reported in this paper.

## Abbreviations

CMR: cardiac magnetic resonance
CP: constrictive pericarditis
CRP: C-reactive protein
CT: computed tomography
CVA4: coxsackievirus A4
CVB: coxsackievirus B
CCT: cardiac computed tomography
ECG: electrocardiogram
ECP: effusive constrictive pericarditis; IL-6, interleukin-6
LGE: late gadolinium enhancement
LIE: late iodine enhancement
NSAIDs: nonsteroidal anti-inflammatory drugs
PCR: polymerase chain reaction
T2WI: T2-weighted image
TCP: transient constrictive pericarditis
