# Supplementary figures and images for: Transient constrictive pericarditis following coxsackievirus A4 infection as a rare cause of acute mediastinitis: A case report
Source: Heliyon. 2023 Aug 30;9(9):e19555. doi: 10.1016/j.heliyon.2023.e19555 (PMC10558803; doi:10.1016/j.heliyon.2023.e19555)

## Slide 1
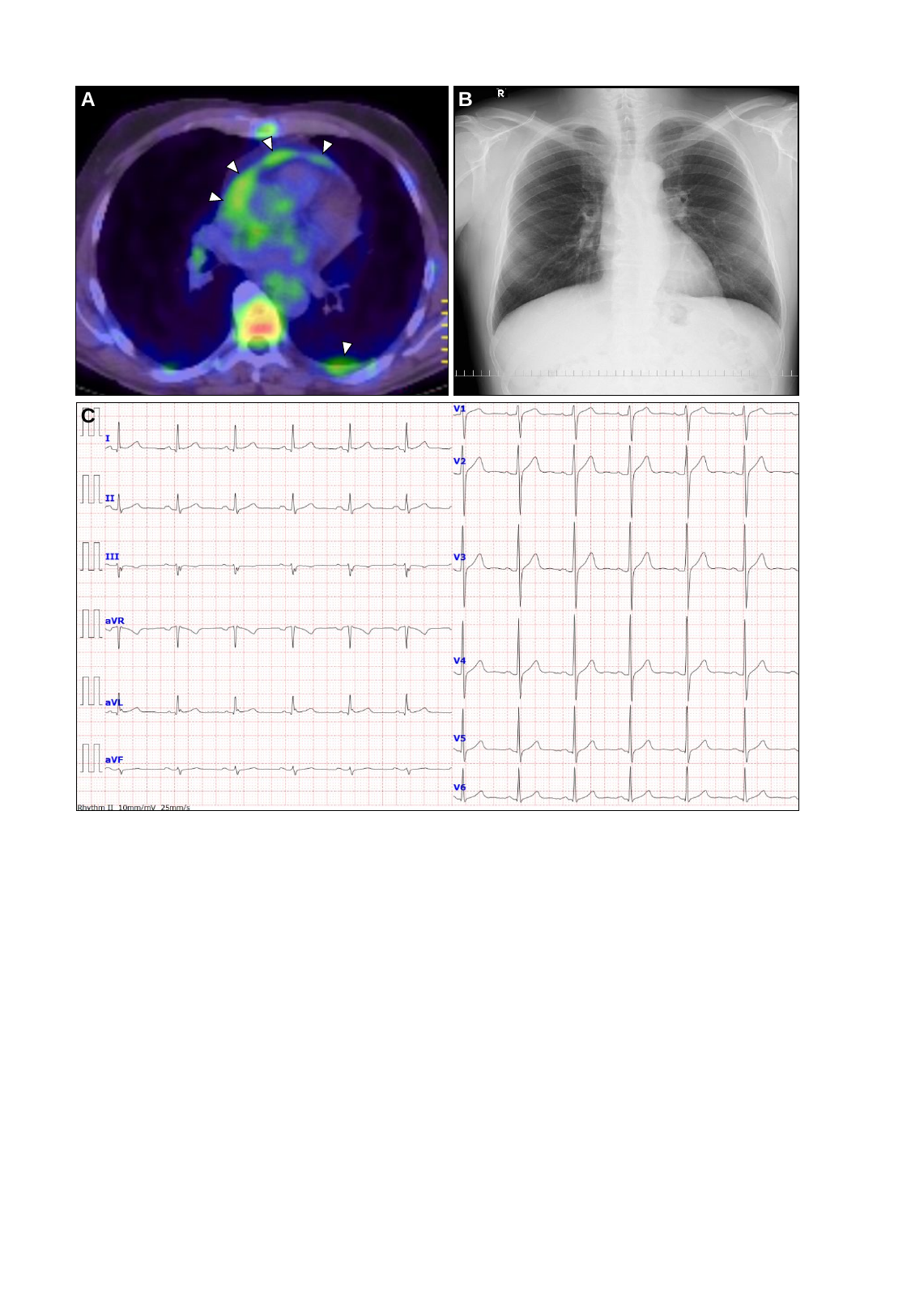

A
B
C

Supplement: Multimedia component 1 [file mmc1.pptx]
